# Molecular typing and drug sensitivity testing of *Mycobacterium tuberculosis* isolated by a community-based survey in Ethiopia

**DOI:** 10.1186/s12889-015-2105-7

**Published:** 2015-08-06

**Authors:** Muluwork Getahun, Gobena Ameni, Abebaw Kebede, Zelalem Yaregal, Elena Hailu, Grimay Medihn, Daniel Demssie, Feven Girmachew, Yetnebersh Fiseha, Abyot Meaza, Nathneal Dirse, Mulualem Agonafir, Feleke Dana, Fasil Tsegaye, Zeleke Alebachew, Almaz Abebe, Amha Kebede, Eshetu Lemma

**Affiliations:** Ethiopian Public Health Institute, Addis Ababa, Ethiopia; Aklilu Lema Institute of Pathobiology, Addis Ababa, Ethiopia; Armauer Hansen Research Institute, Addis Ababa, Ethiopia

## Abstract

**Background:**

The identification of circulating TB strains in the community and drug sensitivity patterns is essential for the tuberculosis control program. This study was undertaken to identify *M. tuberculosis* strains circulating in selected communities in Ethiopia as well as to evaluate the drug sensitivity pattern of these strains.

**Method:**

This study was a continuation of the Ethiopian National TB Prevalence Survey that was conducted between 2010 and 2011. Culture-positive isolates of *M. tuberculosis* from previous study were typed using region of difference (RD) 9-based polymerase chain reaction (PCR) and spoligotyping. Drug sensitivity testing was conducted using the indirect proportion method on Lowenstein-Jensen media.

**Result:**

All 92 isolates were confirmed as *M. tuberculosis* by RD9-based PCR and spoligotyping of 91 of these isolates leds to the identification of 41 spoligotype patterns. Spoligotype revealed higher diversity (45 %) and among this 65.8 % (27/41) were not previously reported. The strains were grouped into 14 clusters consisting of 2–15 isolates. The dominant strains were SIT53, SIT149 and SIT37 consisting of 15, 11, and 9 isolates, respectively. Our study reveals 70 % (64/91) clustered strains and only 39.1 % (25/64) occurred within the same Kebele. Further assignment of the strains to the lineages showed that 74.7 % (68/91) belonged to Euro-American lineage, 18.6 % (17/91) to East Africa Indian lineage and the remaining 6.5 % (6/91) belonged to Indo-oceanic lineage. Valid drug susceptibility test results were available for 90 of the 92 isolates. Mono-resistance was observed in 27.7 % (25/90) and poly-resistance in 5.5 % (5/90) of the isolates. Moreover, multi-drug resistance (MDR-TB) was detected in 4.4 % of the isolates whilst the rest (60/90) were susceptible to all drugs. The highest level of mono-resistance, 26.6 % (24/90), was observed for streptomycin with majority (91.1 %) of streptomycin mono-resistant strains belonging to the Euro-American lineage.

**Conclusion:**

In this study, the strains of *M. tuberculosis* circulating in selected sites of Ethiopia were identified along with the drug sensitivity patterns. Thus, these findings are useful for the TB Control Program of the country.

## Background

There are an estimated 9 million new cases of TB every year. TB is the second leading cause of death from an infectious disease worldwide and is responsible for 1.5 million deaths annually. Control of TB is becoming a challenge to the endemic countries due to the emergence of drug resistance. Recently, the WHO Global TB Report estimated that total of 480,000 people developed MDR-TB worldwide in 2013. The prevalence of MDR-TB has been estimated to be 3.5 % in newly diagnosed cases and 20.5 % in previously treated TB cases [1].

In Ethiopia, TB is a major public health problem and the WHO categorizes Ethiopia as a high TB and MDR-TB burden country [1]. The first Ethiopian National TB prevalence survey was conducted between 2010 and 2011. The prevalence of smear-positive TB among persons aged ≥ 15 years was 108/100,000 (95 % CI 73–143), whereas the prevalence of bacteriologically confirmed TB in the same age group was 277/100,000 (95 % CI 208–347) [2]. According to the nationwide drug resistance survey conducted between 2003 and 2005 the overall prevalence of MDR-TB was 2.5 %. The prevalence of MDR-TB was 1.6 % and 11.8 % in newly diagnosed TB cases and previously treated cases, respectively [3].

Knowledge on the *Mycobacterium tuberculosis* (*M. tuberculosis*) strains circulating in Ethiopian communities and evaluation of the drug sensitivity patterns of these strains is useful for the TB control program in the country. Since the study was done on positive cultures isolated from community-based survey, those patients who have low health seeking behavior were mostly captured. Therefore, this study was undertaken for the identification of the *M. tuberculosis* strains circulating in selected communities in Ethiopia and thereafter to evaluate the drug sensitivity pattern of these strains.

## Methods

### Study population

This study was a continuation of the Ethiopian National TB Prevalence Survey that was conducted between 2010 and 2011. The participants for the prevalence survey carried out by Kebede AH and colleagues were recruited from 85 Kebeles (communes), selected from 85 Woredas (districts) which were stratified as urban, rural and pastoralist population. The number of Kebeles covered in rural, urban and pastoralist areas were 63, 14 and 8 respectively. Sample size determination for the prevalence survey is reported elsewhere [2]. The planned sample size for prevalence survey was 46,514 adults aged ≥15 years. Following this 85 Woredas were selected by multistage cluster sampling using probability proportion to size (PPS) from three strata (urban, rural and pastoralist). In each selected districts one Kebele with estimated eligible adult of 548 was selected using PPS. However during the recruitment of participants the total numbers of individuals from 85 selected clusters were 51,667. Since cluster sampling was used and to maximize sample size, all eligible individuals were invited to participate in the study. Of the total eligible individuals, 46,697 (90 %) participated in the study. The case enrollments for the study were based on symptoms suggestive of TB (cough ≥2 weeks) and/or any abnormality in the lung detected using chest X-ray. For individuals exempt from chest X-ray who did not have cough ≥2 weeks, one of the following criteria was used: weight loss >3 kg in the last 1 month, night sweats >2 weeks, fever >2 weeks and contact with a TB patient in the last year. Using the above criteria a total of 6080 (13 %) participants were eligible for sputum examination. Of these, 5868 (97 %) submitted at least one sputum specimen and 5606 (92 %) submitted two specimens [2]. The present study was carried out on culture positive isolates identified from the prevalence survey.

### Spatial distribution of the study sites at Woreda level

**Fig. 1:**
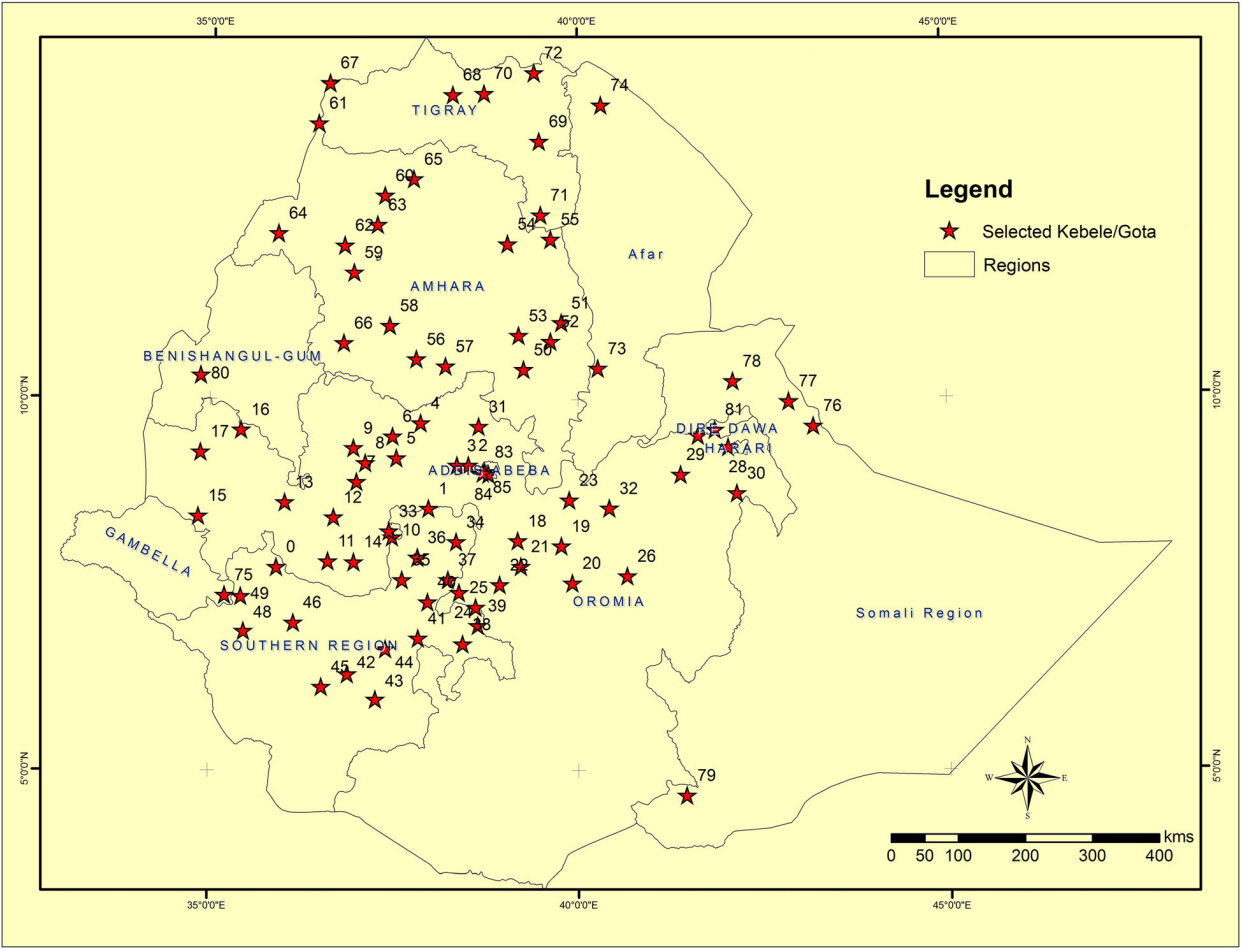
Spatial distribution study sites at Woreda level

 The spatial distributions of the selected study sites are displayed at Woredas level (Fig. 1).

### Laboratory tests

The laboratory analysis was carried out from July to December 2012. For this study, deletion typing, spoloigotyping and drug susceptibility testing were done on positive isolates. Culture and identification of the isolates for the prevalence survey were performed using 4 % NaOH and Capilia test respectively.

### Culture and identification

Equal amounts of 4 % NaOH were added to sputum in a 50 ml screw capped tube. The mixture was vortexed for 1–2 min and kept for 15 min at room temperature. Sterile phosphate buffer (pH =6.8) was added up to 45 ml and then centrifuged at 4 °C for 15 min at 3000 g. After decanting the supernatant, a few drops of sterile phosphate buffer (pH =6.8) was added to reconstitute the sediment. An aliquot of 100–200 μl of the sample sediment was then inoculated into two bottles of Löwenstein- Jensen (LJ) media. The inoculated media was incubated at 37 °C. Bacterial growth was checked for contamination and fast growers in first week and followed weekly up to 8 weeks. Cultures with no growth until the eighth weeks were recorded as negative [4]. Species identification was done for positive LJ cultures. Identification of *M. tuberculosis* complex from other mycobacterial species was performed using Capilia test. The test is based on the *M. tuberculosis* antigen MPB64, which is specific to the members of the *M. tuberculosis* complex. Testing and interpretation of the assays was done according to the manufacturer instruction [5].

### Molecular characterization of the isolates

#### Identification

Identification of *M. tuberculosis* from the other members of the *M. tuberculosis complex* species was done using region of difference (RD)-9 polymerase chain reaction. RD9-PCRdeletion typing was performed on heat-killed cells to confirm the presence or absence of RD9 using three primers RD9flankF, RD9IntR and RD9flankR [6]. PCR amplification was done as indicated by Berg *et al.* using a commercially available kit (Qiagen, United Kingdom). Interpretation of the result was based on differences in molecular weight. A molecular weight of 396bp was considered as *M. tuberculosis* or *M. canettii*, while a molecular weight 575bp was considered *M. bovis* or *M. africanum* [7].

#### Molecular typing

Strain typing of *M. tuberculosis* was done using spoligotyping as previously described by Kamerbeek and colleagues [8]. The PCR amplification of DNA was done by targeting the direct repeat (DR) locus, the region with the highest level of polymorphism in the *M. tuberculosis* chromosome. The DR region was amplified using primers DRa (GGTTTTGGGTCTGACGAC) and DRb (CCGAGAGGGGACGGAAAC). Spoligotyping was performed with a commercially available kit according to the manufacturer instructions (Isogen Bioscience BV Maarssen, The Netherlands). Lineage and SIT (Shared International Types) number assignment was done using SPOLD4 and SPOTCLUST [9].

#### Drug Susceptibility testing

Drug susceptibility testing (DST) was performed for first line drugs; isoniazid, streptomycin, rifampicin and ethambutol, using indirect proportion method on Lowenstein–Jense media. The critical concentration for each drug was 0.2μg/ml, 4μg/ml, 40μg/ml and 2μg/ml for isoniazid, streptomycin, rifampicin and ethambutol respectively. Result interpretation was done by comparing growth on control media and media containing drug. If more than 1 % of the test population was observed on the drug containing media, the result was interpreted as resistant to that drug [10]

#### Statistical analysis

Statistical analysis was carried out using SPSS software packages. Patient characteristics for differences in proportions were compared. The chi square test was used to assess statistical significant difference. *P* values of 0.05 or less were regarded as significant.

#### Ethical consideration

The laboratory analysis was done on stored culture positive samples. Specific patient identifiers were not used for this study. Ethical clearance was obtained from Scientific and Ethics Review Committee at Ethiopian Public Health Institute.

## Result

### Background characteristics

The mean age of culture positive participants was 34.4 years (95 % CI 31.2–37.7). The overall culture positivity rate was 1.6 % (96/5863). The distribution of culture positivity across the categories of background characteristics of TB suspects is summarized in Table 1. The culture positivity was significantly associated with age and region of the study participants. High culture positivity rate (3.2 %) was observed in age group between 15 and 24. Among three regions with high culture positivity rate (Addis Ababa, SNNPR and Gambela) the contribution of younger age categories (15–34) was 44 % (4 out of 9), 60 % (18 out of 30) and 80 % (4 out of 5) from the total positive culture from those regions.

**Table 1:** Culture result Vs sex, age, region and resident type

		Culture Positive	Culture Negative/NTM/Contaminated	Total	Culture Positivity Rate	*P*-value
Sex	Male	51	3122	3173	1.6	0.844
	Female	45	2645	2690	1.7	
Age	15–24	32	962	994	3.2	<0.001
	25–34	21	1183	1204	1.7	
	35–44	17	1084	1101	1.5	
	45–54	12	992	1004	1.2	
	55–64	10	736	746	1.3	
	> = 65	4	810	814	0.5	
Region	Oromiya	28	2250	2278	1.2	<0.001
	SNNPR	30	1191	1221	2.5	
	Amhara	10	948	958	1	
	Tigray	6	505	511	1.2	
	Afar	1	143	144	0.7	
	Gambela	5	83	88	5.7	
	Somali	7	388	395	1.8	
	Benshangul	0	25	25	0	
	Dire Dawa	0	45	45	0	
	Addis Ababa	9	189	198	4.5	
Resident	Urban	14	744	758	1.8	0.844
	Rural	72	4349	4421	1.6	
	Pastoral	10	674	684	1.5	

### Molecular characterization of the isolates

#### Identification

Out of 96 isolates positive for *M. tuberculosis* complex based on Capilia TB immunochromographic assay, 4 samples were excluded from RD9-PCR analysis (2 isolates failed to grow after re-culturing and 2 cultures were not retrieved). The remaining 92 isolates were analyzed based on region of difference analysis. RD9 analysis showed that 92 isolates contained an intact RD9. This indicated that the 92 isolates were *Mycobacterium tuberculosis* species or *M. canettii*.

#### Molecular typing

**Fig. 2:**
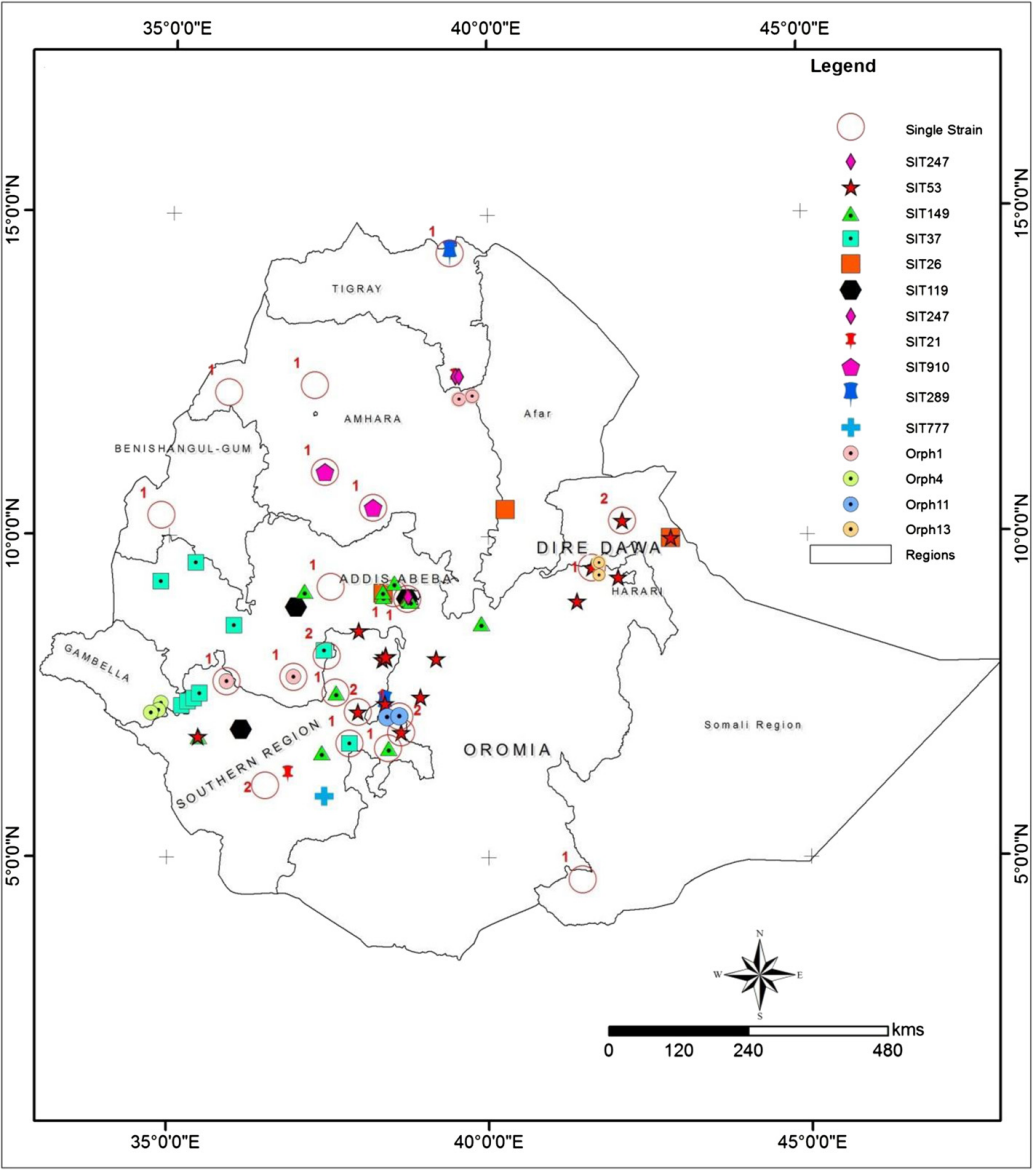
Distribution of strains in their respective collection sites at Woreda level: Both registered and orphan clustered strains were indicated in colored. Orphans were given a number according to their order of identification. Both registered and orphan single isolates is represented in red hallow circle. The numeral in the side of the circle indicates that the number of single strains identified in that specific location

Ninety two isolates were characterized using spoligotyping and 91 of them showed good patterns. Spoligotyping identified 41 different spoligotypes with the overall diversity of 45 % and among this 65.8 % (27/41) were not previously reported. The isolates were grouped into 14 clusters consisting of 2–15 isolates each. The dominant strains of *M. tuberculosis* were SIT53 (15/91), SIT149 (11/91) and SIT37 (9/91). Our study reveals 70 % (64/91) clustered strains of which 53 were registered in the international database whereas 11 were newly identified. Cluster formation within Kebeles was observed only 39.1 % (25/64) (Table 2). The proportion of Kebeles having cluster isolates within the same Kebeles in urban, rural and pastoralist were 28.5 % (4/14), 13.4 % (9/67) and 14.2 % (1/7) respectively. Geographic Information System (GIS) mapping of cluster position of the strain showed that clustering location was far apart (Fig. 2). Among 27 single strains 40.7 % were registered in the international database. Further assignment of the strains to the lineages showed that 74.7 % (68/91) belonged to Euro-American lineage, 18.6 % (17/91) belonged to East Africa Indian lineage and 6.5 % (6/91) to Indo-oceanic lineage.

**Table 2:** Distribution of previously registered and newly identified cluster in Kebeles

	Same Kebele	Different Kebele
SIT for previously registered strains	Number (%)	Number (%)
53	4 (26.7)	11 (73.3)
149	4 (36.4)	7 (63.6)
37	4 (44.4)	5 (55.6)
26	0 (0.0)	3 (100)
119	0 (0.0)	3 (100)
910	0 (0.0)	3 (100)
247	2 (66.7)	1 (33.3)
21	0 (0.0)	2 (100)
289	0 (0.0)	2 (100)
777	2 (100)	0 (0.0)
Sub Total	16 (30.2)	37 (69.8)
Newly identified strains		
Orphan	2 (50.0)	2 (50.0)
Orphan	3 (100)	0 (0.0)
Orphan	2 (100)	0 (0.0)
Orphan	2 (100)	0 (0.0)
Sub Total	9 (81.8)	2 (18.2)
Total	25 (39.1 %)	39 (60.9 %)

#### Drug susceptibility test (DST)

Valid DST results for all first line anti-TB drugs were available for 90 isolates (Table 3). Two isolates were excluded because of contamination. The drug susceptibility pattern of the isolates revealed that 66.6 % (60/90) of the isolates were susceptible to the four first-line drugs tested (streptomycin, isoniazid, rifampicin and ethambutol). Mono-resistance to any one of the four first line drugs tested was found in 27.7 % (25/90) of the cases and poly-resistance was found in 5.5 % (5/90) of cases. The highest level of mono-resistance was observed for streptomycin 26.6 % (24/90). Of the 24 streptomycin mono-resistant strain majority (91.1 %) belonged to the Euro-American lineage and the rest belongs to Indo-oceanic lineage. Multi-drug resistance (MDR) was detected in 4.4 % (4/90) of the cases. The overall MDR-TB prevalence in national survey conducted between 2003 and 2005 was 2.5 % [2]. The prevalence of MDR-TB did not show any significant increase compared to the first round drug resistance survey conducted between 2003 and 2005 (*P* = 0.287). Among the four MDR-TB detected, three of them were isolated from young patients (age ranges 16–20). The lineage of those MDR TB cases showed that three of them belonged to East Africa Indian lineage and one belonged to the Euro-American lineage.

**Table 3:** Susceptibility pattern of 90 isolate for first line drugs

	Number	Percentage
Susceptible	60	66.6
Mono-resistance	25	27.7
Resistant to		
INH only	1	1.1
SM only	24	26.6
INH + SM	1	1.1
MDR	4	4.4
INH + SM + RIF	1	1.1
INH + SM + RIF + EMB	3	3.3

## Discussion

Tuberculosis (TB) is the most common opportunistic infection in HIV positive patients and TB can occur at any time during the course of HIV infection [11]. This study’s findings showed high culture positivity among the younger age groups and in three regions. The TB/HIV sentinel report from 2010 to 2014 indicated that out of those three regions, two regions ranked as the two most HIV prevalent among TB patients (unpublished data), which may account for the higher culture positivity rate in younger age groups in those regions.

Most of human TB is caused by *M. tuberculosis* although some cases are caused by *M. bovis*. In this study, RD9deletion typing was used to differentiate between *M. bovis* and *M. tuberculosis* [12, 13]. Even though most of the isolates were collected in rural and pastoralist population where raw milk consumption is widely practiced, all the isolates obtained by this study were *M. tuberculosis*. The absence of *M. bovis* among the isolates collected from rural communities was unexpected and contradicts with the earlier studies [14, 15]. The r absence of *M. bovis* requires further study and may be due to the manifestation of *M. bovis* in extra-pulmonary TB.

Cluster formation and epidemiological links have been used to qualify recent transmission [16]. Our finding reveals clustering within same Kebele was low (39.1 %) even without considering the parameters for making an epidemiological link. Since the larger portions of our samples were collected from rural populations that were sparsely populated, this might result in low rate of transmission as a result clustering rate will be decreased, which is in line with previous findings [16, 17]. Branden and colleagues suggested that the transmission of common endemic strains of *M. tuberculosis* occurs in relatively closed populations but in a rural setting the presence of clustering often is not associated with recent transmission; it could be the result of reactivation of remote infections acquired years or decades earlier.

Understanding the prevalence of TB drug resistance offers an opportunity to assess the extent of resistant bacteria transmission in the community as well as evaluate effect of the National Tuberculosis Program [18]. The MDR rate did not show any significant increment compared to the previous nationwide surveillance report even though the study population was different [3]. A study conducted in the Philippines at hospitals in Metro Manila, multi- drug resistant TB prevalence rates in hospital ranged from 14–45 %, whereas the community rate of MDR TB in Metro Manila was 6.4 % [19]. This suggested that high rate of MDR TB occur in health facilities. Thus in Ethiopia, the rate of MDR TB in the health facilities might be higher than the finding of this current study. Out of 4 MDR cases identified in the present study, three MDR TB cases occurred in participants 16–20 years old. As reported by another study [20] the high prevalence of primary MDR-TB in a young population group implies transmission of drug resistant strains.

## Conclusions

In this study, the strains of *M. tuberculosis* circulating in selected sites of Ethiopia were identified along with the drug sensitivity patterns. More than 83 % of the population is living in rural areas of Ethiopia. Since declining incidence of TB is expected with decreased clustering, coupling case detection and treatment alone in those large portions of the rural population will have a huge contribution to the reduction of TB. Besides, TB and MDR rate in young was high but with such small number of cases the possible relationship is inconclusive it needs further study.
